# Cognitive impairment in Parkinson's disease: impact on quality of life of carers

**DOI:** 10.1002/gps.4623

**Published:** 2016-12-07

**Authors:** RA Lawson, AJ Yarnall, F Johnston, GW Duncan, TK Khoo, D Collerton, JP Taylor, DJ Burn, On behalf of the ICICLE‐PD study group

**Affiliations:** ^1^ Institute of Neuroscience Newcastle University Newcastle upon Tyne UK; ^2^ Centre for Clinical Brain Sciences University of Edinburgh Edinburgh UK; ^3^ School of Medicine and Menzies Health Institute Queensland Griffith University Queensland Australia

**Keywords:** Cognitive impairment, Parkinson's disease, quality of life, carer

## Abstract

**Background:**

The quality of life (QoL) of informal caregivers of people with Parkinson's disease (PD) (PwP) can be affected by the caring role. Because of cognitive symptoms and diminished activities of daily living, in addition to the management of motor symptoms, carers of PwP and cognitive impairment may experience increased levels of burden and poorer QoL compared with carers of PwP without cognitive impairment. This study aimed to investigate the impact of cognitive impairment in PD upon QoL of carers.

**Methods:**

Approximately 36 months after diagnosis, 66 dyadic couples of PwP and carers completed assessments. PwP completed a schedule of neuropsychological assessments and QoL measures; carers of PwP completed demographic questionnaires and assessments of QoL. Factor scores of attention, memory/executive function and global cognition, as derived by principal component analysis, were used to evaluate cognitive domains.

**Results:**

Hierarchical regression analysis found lower Montreal Cognitive Assessment was a significant independent predictor of poorer carer QoL, in addition to number of hours spent caregiving, carer depression and PD motor severity. Attentional deficits accounted for the largest proportion of variance of carer QoL. Carers of PwP and dementia (*n* = 9) had significantly poorer QoL scores compared with PwP and mild cognitive impairment (*n* = 18) or normal cognition (*n* = 39) carers (*p* < 0.01).

**Conclusions:**

Attentional deficits were the strongest predictor of carer QoL compared with other cognitive predictors. Carers for those with PD dementia reported the poorest QoL. Interventions such as respite or cognitive behavioural therapy to improve mood and self‐efficacy in carers may improve carer QoL. © 2016 The Authors. *International Journal of Geriatric Psychiatry* published by John Wiley & Sons, Ltd.

## Introduction

Informal carers are a crucial source of support for individuals with Parkinson's disease (PD), providing both physical and emotional support. It is estimated that informal carers of people with PD (PwP) save, for example in the UK, over £70 000 per person annually in health and social care costs (Jackson *et al.,* 2013) compared with formal support. However, the quality of life (QoL) of these informal caregivers of PwP is affected by the caring role (Goldsworthy and Knowles, 2008). Carers of PwP are often spouses who are themselves elderly with and may have their own health problems (Berry and Murphy, 1995). Increased carer strain has previous been associated with early nursing home placement for PwP (Abendroth *et al.,* 2012; Goetz and Stebbins, 1993).

Quality of life is subjective and difficult to define (Martinez‐Martin, 1998); it includes physical, psychological and social elements as well as personal and cultural context (World Health Organization, 1993). Poor QoL can lead to poor psychosocial consequences. PD motor severity, falls and neuropsychiatric symptoms in PwP predict poorer QoL in carers (Aarsland *et al.,* 1999; Abendroth *et al.,* 2012; Santos‐Garcia and de la Fuente‐Fernandez, 2015), reduced social activities, financial strain, perceived strain and emotional health have been associated with poorer carer QoL (Aarsland *et al.,* 1999; Leiknes *et al.,* 2010; Schrag *et al.,* 2006). Previous studies have shown a relationship between QoL in PwP and the QoL in informal carers (Miyashita *et al.,* 2011; Peters *et al.,* 2011). Poorer PD QoL may be a primary stressor for QoL in their carer. Alternatively, there may be a mutually dependent relationship between carers and PwP, where the biopsychosocial functioning in one person is dependent on the same underlying features in the other person (Greenwell *et al.,* 2015).

Thommessen *et al.* (2002) suggested that carers of patients with dementia and carers of PwP experience similar levels of burden. However, it may be that the carers of PwP and cognitive impairment, in addition to caring for motor and non‐motor symptoms in PwP, experience an increased burden. For example, carers of PwP and cognitive impairment may acquire additional and unfamiliar household responsibilities as PwP experience memory problems and challenges in organizing and decision making, plus personal and domestic activities of daily living because of disability. They may also have had to cope with the emotional impact of watching their partner or relative decline both physically and cognitively (Carter *et al.,* 2012). Previous studies have shown that increased carer burden is greater in PD dementia carers (PDD) carers compared with those with mild cognitive impairment (PD‐MCI) (Leroi *et al.,* 2012) and that carer burden is associated with behavioural problems related to executive dysfunction (Kudlicka *et al.,* 2014). Furthermore, neuropsychiatric symptoms are more common in PDD and have also been associated with increased carer burden (Oh *et al.,* 2015).

This study investigated whether cognitive impairment in PwP was an additional stressor to QoL. Furthermore, we investigated whether impairment in particular cognitive domains in PwP were associated with poorer carer QoL. This could be potentially useful to guide clinicians as to appropriate interventions to protect carer QoL, which could lead to delays in formal care and nursing home placement.

## Methods

### Participants

This study is part of the incidence of cognitive impairments in cohorts with longitudinal evaluation in Parkinson's disease (ICICLE‐PD) study (Yarnall *et al.,* 2014). Newly diagnosed PD patients from community and outpatient clinics in Newcastle upon Tyne and Gateshead were invited to take part in the study between June 2009 and December 2011. After baseline assessments, participants were invited back at 18‐month intervals where PwP were subsequently re‐assessed at approximately 18 and 36 months (Lawson *et al.,* 2016). Idiopathic PD was diagnosed by a movement disorder specialist and fulfilled Queen's Square Brain Bank criteria (Hughes *et al.,* 1992). Participants were excluded from ICICLE‐PD at baseline if they had significant cognitive impairment at initial assessment (mini‐mental state examination (MMSE) <24) or a diagnosis of dementia (Emre *et al.,* 2007). Age‐sex matched healthy control subjects (*n* = 99) were recruited to provide normative data, which has been previously reported by Yarnall *et al.* (2014).

Carer assessments were added to the 36‐month evaluation to investigate the wider effects of PD and cognitive impairment. Informal carers of PwP who returned at 36 months were invited to complete additional carer assessments (*n* = 66). Informal carers were spouses, partners, adult family members or friends who were the primary caregiver of PwP. The study was approved by the Newcastle and North Tyneside Research Ethics Committee. All subjects provided written informed consent.

### Carer assessments

Demographic information of carers was collected, including age, sex, education and hours per week caregiving. The Hospital Anxiety and Depression Scale (HADS) subscales (Zigmond and Snaith, 1983) were used to measure anxiety and depression; higher scores indicated more severe symptoms. Neuropsychiatric symptoms and carer distress were measured by the Neuropsychiatric Inventory (NPI) and NPI Carer Distress (NPI‐D) (Cummings *et al.,* 1994).

Carer QoL was measured by the Scale of Quality of Life of Care‐Givers (SQLC) (Glozman *et al.,* 1998), which is a PD specific measure of QoL in carers. The scale gives a total score ranging from 4–149; lower scores indicate poorer QoL.

### Clinical and neuropsychological assessments in patients

Patient demographic information, including age, sex and education was collected. PwP completed the Movement Disorder Society (MDS) Unified Parkinson's Disease Rating Scale (UPDRS) Part III (Goetz *et al.,* 2008) and the Geriatric Depression Scale (GDS‐15) (Yesavage *et al.,* 1982). Subjects were assessed in an ‘on’ motor state. Levodopa equivalent dose was calculated for all dopaminergic medications using methods described by Tomlinson *et al*. (2010). The Parkinson's Disease Questionnaire (PDQ‐39) summary index was used as a global measure of QoL in PwP and is well validated (Jenkinson *et al.,* 1997) and is a recommended measure of QoL in PD by the MDS task force (Martinez‐Martin *et al.,* 2011). The scores ranged from 0 (best possible QoL) to 100 (worst possible QoL).

People with Parkinson's disease completed a schedule of neuropsychological tests as described previously (Yarnall *et al.,* 2014). In brief, global cognitive function was assessed using the MMSE (Folstein *et al.,* 1975) and Montreal Cognitive Assessment (MoCA) (Nasreddine *et al.,* 2005). Attention was measured using power of attention and digit vigilance accuracy from the Cognitive Drug Research battery (Nicholl *et al.,* 1995). Memory was assessed using the number of correct answers from pattern recognition memory and spatial recognition memory, and mean trials to success for paired associate learning from the Cambridge Neuropsychological Test Automated Battery (Robbins *et al.,* 1994). Executive function was assessed using the one touch stockings from the Cambridge Neuropsychological Test Automated Battery, phonemic fluency (number of word generated in 60s beginning with the letters F, A and S) and semantic fluency (number of animals generated in 90s). Visuospatial function was evaluated using the pentagon copying item within the MMSE, graded using a modified 0–2 rating scale (Ala *et al.,* 2001). Language was assessed using the naming (0–3) and sentence (0–2) items in the MoCA.

People with Parkinson's disease were classified as having mild cognitive impairment if they performed two standard deviations (SD) below the means of appropriate norms (controls) on at least two neuropsychological tests across the five cognitive domains in addition to subjective cognitive impairment (Litvan *et al.,* 2012). Recent studies have suggested 2 SD may be a suitable cut‐off to distinguish PD‐MCI from Parkinson's disease‐normal cognition (PD‐NC) (Goldman *et al.,* 2013; Lawson *et al.,* 2014). PDD was diagnosed using the MDS criteria (Emre *et al.,* 2007).

### Statistical analysis

Statistical analyses were performed using spss software (Version 22.0; SPSS, Armonk, NY: IBM Corp). Data were examined for normality of distribution with visual histograms and Kolmogorov–Smirnov's test. Comparisons of means between two groups were performed using independent *t*‐tests or Mann–Whitney *U*‐test as required. For more than two group comparisons, one way anovas or Kruskal–Wallis tests were used as appropriate. Multiple comparisons were corrected using Bonferroni's correction. Correlations were assessed using Spearman's rho. Principal component analysis (PCA) using oblique oblimin rotation was used to reduce the large number neuropsychological assessments to a smaller number of cognitive dimensions, which has been previously described by (Lawson *et al.,* 2016). Factor scores were then calculated using the component score coefficient matrix at baseline and 36 months. Hierarchical regression models were used to build predictive models of QoL. Backwards stepwise regression was used to determine a basic model predicting SQLC. Variables included in the model were carer age, gender, years of education, hours per week caregiving, Hospital Anxiety and Depression Scale‐Anxiety subscale and Hospital Anxiety and Depression Scale*‐*Depression subscale. Age of PwP, UPDRS III, Levodopa equivalent dose, GDS‐15, NPI, NPI‐D and PDQ‐39 were also included. Cognitive measures of PwP were then separately added to the model to determine whether cognition was an added stressor to carer QoL.

## Results

At 36 months, 66 dyadic couples of informal carers and PwP carers (*n* = 66) had a mean age of 67 ± 11.5 years with a range of 32 years to 85 years (Table 1). Eighty‐one percent of carers were female, and 70% were retired.

**Table 1:** Carer demographic characteristics

	Mean	*SD*	I‐Q range
Carer age (years)	67.1	11.5	14
Education (years)	12.2	2.8	4
Years known participant	46.2	15.0	15
Time as a carer (months)	23.5	24.5	36
Hours per week as caregiver	50.5	69.1	108
	*n*	%	
Gender, female	55	81	
Relationship to care recipient			
Spouse or partner	63	93	
Daughter	2	3	
Other relative	1	2	
Friend	2	3	
Employment status, retired	41	70	
Other caregiving responsibilities	19	28	
Children	4	6	
Grandchildren	12	18	
Other relative	3	4	

*n* = 57, *n* = 9 coded as missing data: the questionnaire was introduced later in the study (*n* = 4) and not completed by carer (*n* = 5); SD = standard deviation, I‐Q = inter‐quartile range.

### Characteristics of patients with Parkinson's disease

In total, 110 PwP of 158 returned for 36 month evaluation; 44 PwP did not have carers as they were independent or their relatives did not describe themselves as a carer and so did not complete the questionnaires. Significant differences at 36 months between PwP with and without carers are described in Table 2. There was no significant difference in the proportion of PD‐MCI between the two groups, although all PDD subjects had a carer (*n* = 9). Neuropsychological test scores of PwP and carers at baseline and 36 months are described in Table 3. PCA reduced baseline neuropsychological tests into three principal components: memory/executive function (paired associated learning, paired recognition memory, spatial recognition memory and one touch stockings), attention (power of attention and digit vigilance) and global cognition (MMSE, MoCA, verbal fluency and semantic fluency) accounting for 40%, 12% and 10% of the variance, respectively. Factor scores were calculated using baseline and 36‐month data (Table 3).

**Table 2:** Clinical and demographic characteristic of participants with and without carers

	No carer (*n* = 42)		Carer (*n* = 66)		t/Z	*p*
	Mean	*SD*	Mean	*SD*		
Age (years)	66.1	10.6	71.3	9.0	−2.6	**0.010**
MDS‐UPDRS III	37.5	14.7	39.6	12.4	−1.0	0.339
Hoehn and Yahr stage	2.2	0.4	2.2	0.5	0.0	0.984
GDS‐15	2.7	2.7	2.9	2.4	−0.8	0.447
LED (mg/day)	499.1	305.5	526.3	258.8	−0.8	0.411
PDQ‐39 SI	20.7	15.9	23.7	17.1	−0.9	0.363
MoCA	26.3	3.9	25.4	4.0	−1.7	0.095
MMSE	28.3	1.9	27.9	2.7	−0.1	0.887
	*n*	%	*n*	%	*χ* ^2^	*p*
Gender (male)	22	52	50	76	10.9	**0.001**
Marital status						
Married/living with partner	19	45	64	97	24.4	**<0.001**
Widowed	10	24	2	3	5.3	**0.021**
Divorced	6	14	0	0	–	–
Single	7	17	0	0	–	–
ADL, not independent	5	12	24	36	7.8	**0.005**
Cognitive classification						
PD‐MCI	12	29	18	27	1.2	0.273
PDD	0	0	9	14	–	–

MDS‐UPDRS III = Movement Disorders Society‐Unified Parkinson's Disease Rating Scale Part III; GDS‐15 = Geriatric Depression Score; LED = Levodopa equivalent dose; PDQ‐39 SI = Parkinson's Disease Questionnaire Summary Index Score; MoCA = Montreal Cognitive Assessment; MMSE = Mini Mental State Examination; ADL = activities of daily living; PD‐MCI = mild cognitive impairment in Parkinson's disease using two standard deviation cut‐off; PDD = Parkinson's disease dementia; SD, standard deviation.

Significant differences highlighted in bold.

**Table 3:** Neuropsychological test scores of patients with Parkinson's disease and carers at baseline and 36‐month assessments

		Baseline		36 months		Paired differences (36 months‐baseline)		Z	*p*‐value
		Mean	*SD*	Mean	*SD*	Mean	*SD*		
MoCA		25.1	3.6	25.4	4.0	0.3	3.0	−0.4	0.669
MMSE		28.6	1.2	27.9	2.7	−0.7	2.1	−2.3	**0.021**
PoA		1356.5	218.3	1484.8	320.1	119.5	281.0	−4.4	**0.000**
Digit vigilance		91.5	13.9	88.8	15.5	−2.8	12.1	−1.5	0.127
PRM		19.8	2.7	19.2	3.6	−0.6	2.8	−1.8	0.075
SRM		15.5	2.1	13.8	2.5	−1.8	2.6	−4.8	**0.000**
PAL		2.0	0.7	2.4	1.2	0.4	0.9	−3.3	**0.001**
OTS		13.8	3.9	12.2	5.8	−1.6	4.2	−2.6	**0.008**
Phonemic fluency		33.6	11.1	35.1	13.7	1.5	10.3	−1.0	0.321
Semantic fluency		20.6	6.1	19.8	7.4	−0.8	6.7	−1.3	0.184
Factor scores									
	Memory/executive function	0.00	1.09	−0.15	1.13	−0.13	0.68	−0.9	0.373
	Attention	−0.03	0.99	−0.13	0.99	−0.04	0.77	−0.4	0.664
	Global cognition	0.01	0.92	−0.16	1.07	−0.11	0.67	−0.8	0.396

SD = standard deviation; MoCA = Montreal Cognitive Assessment; MMSE = Mini Mental State Examination; PoA = power of attention; PRM = paired recognition memory; SRM = spatial recognition memory; PAL = paired associated learning, OTS = one touch stockings.

Memory/executive function comprises PRM, SRM, PAL and OTS; attention comprises PoA and digit vigilance; global cognition comprises MoCA, MMSE, phonemic fluency and semantic fluency.

Significant differences highlighted in bold.

### Cognitive impairment in Parkinson's disease and quality of life in carers

There was a significant relationship between PDQ‐39 score in the PwP and SQLC scores in the carer (ρ = −0.48, *p* < 0.01) where poorer QoL in PwP was associated with poorer QoL in the carer. Lower MoCA scores in the PwP at 36 months were moderately associated with lower SQLC scores (ρ = 0.40, *p* < 0.01). Examining the factor scores from the PCA analysis at 36 months, SQLC score and attention had the strongest association (ρ = 0.51, *p* < 0.01); memory/executive function (ρ = 0.47, *p* < 0.01) and global cognition (ρ = 0.43, *p* < 0.01) were also significantly correlated.

There were no significant differences between carers in the cognitive groups at 36 months in terms of age, anxiety, depression, sleep quality or physical health (Table 4, *p* > 0.05 for all). There was also no significant difference in carer rated NPI or NPI‐D scores. There was a threefold difference in hours per week caregiving PDD carers compared with PD‐NC or PD‐MCI (Table 4, *p* < 0.01 for both), but not between PD‐NC and PD‐MCI carers. A similar pattern was found for QoL, where between group differences were significant (*p* < 0.05 for both), but SQLC score of PDD carers was significantly higher than PD‐NC and PD‐MCI carers (*p* < 0.01), who did not differ from each other.

**Table 4:** Comparison of carer questionnaires between cognitive groups

	PD‐NC		PD‐MCI		PDD		*χ* ^2^	*p*	
	(*n* = 39)		(*n* = 18)		(*n* = 9)				
	Mean	*SD*	Mean	*SD*	Mean	*SD*			
Carer Age	65.7	13.6	70.2	6.5	67.3	11.2	0.8	0.672	
Time caregiving (hours/week)	37.8	63.0	35.2	61.0	123.6	64.0	10.9	**0.004**	a, b
SQLC	115.6	12.9	109.8	14.2	88.8	21.2	13.1	**0.001**	a, b
HADS‐A	5.2	4.2	4.3	4.5	6.0	5.3	1.0	0.611	
HADS‐D	3.2	2.7	3.1	3.9	6.3	3.8	4.8	0.092	
NPI total	7.5	7.5	9.6	11.6	14.6	13.1	1.7	0.429	
NPI carer distress total	4.0	4.3	5.1	6.6	6.8	6.9	1.0	0.645	
Sleep quality	60.6	21.3	60.9	24.9	52.8	23.3	0.6	0.757	
Total number health problems	2.0	2.0	1.9	1.8	1.6	1.5	0.3	0.880	

Post hoc Bonferroni correction for three group comparisons at *p* < 0.0167; *a* = PD‐NC versus PD‐PDDI; *b* = PD‐MCI versus PDD.

PD‐NC = Parkinson's disease‐normal cognition; PD‐MCI = mild cognitive impairment in Parkinson's disease using the two standard deviation cut‐off; PDD = Parkinson's disease dementia; SD = standard deviation; SQLC = Scale of Quality of Life of Care‐Givers; HADS‐A = Hospital Anxiety and Depression Scale‐Anxiety subscale; HADS‐D = Hospital Anxiety and Depression Scale‐Depression subscale; NPI = Neuropsychiatric Inventory.

Significant differences highlighted in bold.

### Predicting carer quality of life

Backwards stepwise regression showed that increased hours spent caregiving (β = −0.5); carer depression (β = −0.2) and PD motor severity (β = −0.3) predicted poorer SQLC scores (*p* < 0.05 for all). The model accounted for 54% variance (adjusted *R* ^2^ = 0.54, *p* < 0.01).

Cognitive performances of the PwP were individually added to the basic model to determine their contribution to carer QoL. To determine whether global cognitive impairment was an additional stressor to carer QoL, 36‐month MoCA score was added to the basic model (Table 5). Poorer MoCA score of the PwP was a significant predictor of poorer carer QoL and improved the basic model by 4% (∆*R* ^2^ = 0.04, *p* < 0.05).

**Table 5:** Regression coefficients and model fit of cognitive predictors of SQLC scores

	β	*t*	*p*	95% CI for β	
				Lower bound	Upper bound
Basic model† + MoCA^a^	0.3	2.2	**0.030**	0.1	1.9
Basic model† + Factor scores^b^					
Memory/Executive function factor score	0.23	1.54	0.130	−1.03	7.72
Attention factor score	0.25	2.02	**0.049**	0.01	7.97
Global cognition factor score	0.02	0.12	0.906	−3.78	4.25
Basic model† + Attention factor score^c^	0.4	3.9	**<0.001**	3.0	9.5

† Additional covariates in each model = hours caregiving, HADS‐D and UPDRS III.

SQLC = Scale of Quality of Life of Care‐Giver; CI = confidence interval; MoCA = Montreal Cognitive Assessment; HADS‐D = Hospital Anxiety and Depression Scale‐Depression Subscale, UPDRS III = Movement Disorders Society‐Unified Parkinson's Disease Rating Scale Part III; SE, standard error.

a *R* = 0.82, *R* ^2^ = 0.68, adjusted *R* ^2^ = 0.65, *SE* = 10.4, Δ*R* ^2^ = 0.04

b *R* = 0.87, *R* ^2^ = 0.76, adjusted *R* ^2^ = 0.72, *SE* = 9.3, Δ*R* ^2^ = 0.11

c *R* = 0.86, *R* ^2^ = 0.73, adjusted *R* ^2^ = 0.71, *SE* = 9.5, Δ*R* ^2^ = 0.09

Significant differences highlighted in bold.

To determine whether impairments in specific domains predicted carer QoL, factor scores at 36 months were added to the basic model (Table 5). The factor scores accounted for 11% of the total variance (∆*R* ^2^ = 0.11, *p* < 0.01); however, only attention was a significant predictor (*p* < 0.05). Repeating the analysis with memory/executive function and global cognition removed as non‐significant factors, showed poorer attention scores in PwP predicted poorer carer QoL and accounted for 9% of the total SQLC variance (∆*R* ^2^ = 0.09, *p* < 0.01).

### Baseline cognition and carer quality of life at 36 months

As a secondary analysis, we examined the association between baseline cognition in PwP and carer QoL at 36 months. SQLC score was moderately correlated with baseline MoCA score (ρ = 0.34, *p* < 0.05), baseline memory and executive function (ρ = 0.48, *p* < 0.01) and baseline attention (ρ = 0.40, *p* < 0.01), but not baseline general cognition (ρ = 0.16, *p* > 0.05). Regression analysis using baseline predictors of PwP was performed; significant predictors in the basic model were PwP age (β = −0.5), UPDRS III score (β = −0.9) and GDS‐15 score (β = 1.2). The model accounted for 34% variance (adjusted *R* ^2^ = 0.34, *p* < 0.01). Baseline MoCA score was a significant predictor of future SQLC score (∆R^2^ = 0.09, β = 1.6, *p* < 0.01). Baseline factor scores were not significant predictors of SQLC at 36 months (memory and executive function β = 3.7; attention β = 3.2; general cognition β = 2.4; *p* > 0.05 for all).

## Discussion

This is the first study to investigate the impact of cognitive function in PwPs upon carer QoL in a large incident group. This study demonstrated that cognitive impairment in PwP was an additional stressor to carer QoL and that impaired attention in particular contributed to carer QoL. Previous studies have found associations between cognition and carer burden (Kudlicka *et al.,* 2014; Leroi *et al.,* 2012; Martinez‐Martin *et al.,* 2008), which contributes to QoL. A study by Martínez‐Martín *et al.* (2005) found a correlation between cognitive state, as measured by the Short Portable Mental State Questionnaire, and carer QoL, albeit with less comprehensive neuropsychological assessments.

Patient MoCA score, a frequently used clinical measure of global cognition, was a significant predictor of carer QoL, indicating that carers of PwP who scored poorly on the MoCA were more likely to report worse. Previous studies have shown poorer cognition, as measured by the MMSE, in PwP to be associated with poorer physical health of their carer, increased stress and higher psychosocial burden (Aarsland *et al.,* 1999). However, such measures may not be a sufficiently sensitive to capture the different domains of cognitive impairment in PD (Litvan *et al.,* 2012).

There is a paucity of studies investigating whether cognitive phenotype in PwP may affect carer QoL. Using PCA analysis, we found that factor scores for attention, memory/executive function and global cognition were also significantly associated with carer QoL. However, only poorer attention was a significant predictor of carer QoL; this suggests that attentional deficits may play a role in reduced carer QoL. It has been suggested that attentional deficits of PDD patients are detrimental to basic and instrumental activities of daily living in PwP (Bronnick *et al.,* 2006). This includes physical activities, as well as social interactions including participating in conversations, keeping appointments and engagement in leisure activities. Speculatively, carers may have to compensate for the effects of attentional impairments in PwP by increasing their caring responsibilities, which may reduce their QoL (Abendroth *et al.,* 2012).

This study demonstrates that QoL among carers was worse for those who cared for someone with PDD compared with carers of an individual who was deemed to be PD‐NC or PD‐MCI. No significant differences in carer QoL scores were observed between carers for those with PD‐NC and PD‐MCI. These results are comparable with the findings of Leroi *et al.* (2012), who reported that carer burden was significantly higher in PDD carers compared with PD‐NC or PD‐MCI carers, although they did not assess carer QoL.

Almost two‐thirds of PwP in this study had an informal carer at their 36‐month evaluation. Those without carers tended to be younger, female and had no spouse or partner (widowed, divorced or single). Carers tended to be spouses and were predominantly women in their late 60s. The demographics of carers and care recipients in this study are similar to previous reports (Glozman, 2004; Peters *et al.,* 2011; Santos‐Garcia and de la Fuente‐Fernandez, 2015).

Carer QoL was also significantly associated with PD disease motor severity and the number of hours spent caregiving per week. Severity of PD has been associated with carer burden and strain in previous studies (Santos‐Garcia and de la Fuente‐Fernandez, 2015), but not directly to carer QoL, as in our study. The number of hours spent caregiving has previously been reported to affect carer well‐being and distress, while increased frequency of carer breaks may be protective (Goldsworthy and Knowles, 2008). Carer depression was predictive of poorer carer QoL; previous studies have generally focussed on depression in the PwP, with only a few researchers evaluating the effect of depression among carers (Martínez‐Martín *et al.,* 2007). Therefore, these results highlight the importance of assessing depressive symptoms in the carer, as well as in the PwP.

The strengths of this study include the range of validated instruments, the comprehensive schedule of neuropsychological tests and the variety of carer measures. A minority of carers did not complete all measures, which may have reduced the power of the statistical analysis. Thus, subtle differences or associations may not have been detected. The cross‐sectional nature of this study was also a limitation; as these measures were introduced approximately 3 years after diagnosis, no causal relationships could be determined. Although the SQLC was designed for carers of PwP and has good internal consistency (Glozman *et al.,* 1998), QoL is difficult to measure because of variation in definitions and subjectivity between individuals (Glozman, 2004). PD‐MCI criteria have not yet been validated; we used a more conservative 2 SD cut‐off to increase diagnostic certainty of PD‐MCI, although previous studies have shown that both 1.5 SD and 2 SD are suitable (Goldman *et al.,* 2013; Wood *et al.,* 2016). Furthermore, some PwP did not return for the 36‐month evaluation; these may have been of greatest interest as their carers may experience more strain, burden or poorer QoL. Baseline motor severity of PwP was poorer in participants who did not return at 36 months; however, there were no other significant differences between those who returned and those who did not (Supplementary Table 1). Finally, protective factors were not accounted for in this study which may mitigate the negative factors, for example coping styles (Greenwell *et al.,* 2015).

## Conclusion

This study demonstrates an association between cognitive impairment in PwP and poorer carer QoL. Carers of those with PDD reported the poorest QoL. Attentional deficits in PwP were the strongest predictor of carer QoL compared with other cognitive predictors. Our results suggest that respite to reduce the number of hours per week spent caregiving may be readily implemented means of improving carer QoL. However, respite care can be difficult to access because of low availability and reduced funding (Laverty *et al.,* 2016). Interventions to improve cognition in PwP may improve both patient (Lawson *et al.,* 2016) and carer QoL. Carers of PwP and cognitive impairment may benefit from cognitive behavioural therapy or acceptance and commitment therapy, which have been effected for dementia caregivers (Losada *et al.,* 2015). Further research is needed to substantiate these findings, particularly to determine the longitudinal impact and potential protective factors of carer QoL. Longitudinal studies should also consider the cognitive changes in carers and consider the impact this has on both the carer and the PwP, and other outcomes such as nursing home placement.

### Funding

Incidence of cognitive impairments in cohorts with longitudinal evaluation in Parkinson's disease was funded by Parkinson's UK (J‐0802) and Lockhart Parkinson's Disease Research Fund. The research was supported by the National Institute for Health Research (NIHR) Newcastle Biomedical Research Unit based at Newcastle upon Tyne Hospitals NHS Foundation Trust and Newcastle University.

**Key points**

- Cognitive impairment in people with Parkinson's disease is associated with poorer carer quality of life.
- Attentional deficits in people with Parkinson's disease were the strongest predictor of carer quality of life compared with other cognitive predictors.
- Respite and cognitive behavioural therapy to improve mood and self‐efficacy in carers may improve carer quality of life.

## Supporting information

Supporting info item
