# Supplementary material for: Cognitive impairment in Parkinson's disease: impact on quality of life of carers
Source: Int J Geriatr Psychiatry. 2016 Dec 7;32(12):1362–70. doi: 10.1002/gps.4623 (PMC5724657; doi:10.1002/gps.4623)
Supplement: Supplementary file 1 — Supporting info item [file GPS-32-1362-s001.docx]

Supplementary Table 1: Comparison of baseline demographic and clinical characteristics of participants who returned for 36 month evaluation (completers) and non-completers of assessments

|  | Completers (n=110) | | Non-completers (n=44) | | t/Z | p |
| --- | --- | --- | --- | --- | --- | --- |
|  | Mean | SD | Mean | SD |  |  |
| *Age (years)* | 66.1 | 9.9 | 67.1 | 11.6 | -0.6 | 0.561 |
| *Education (years)* | 12.9 | 3.6 | 12.4 | 4.2 | -1.0 | 0.297 |
| *NART* | 114.6 | 11.0 | 115.0 | 9.9 | 0.0 | 0.993 |
| *PD duration (months)* | 6.1 | 4.5 | 7.0 | 8.9 | -0.3 | 0.787 |
| *MDS-UPDRS III* | 25.4 | 10.8 | 30.8 | 14.3 | -2.0 | 0.044 |
| *Hoehn and Yahr stage* | 1.9 | 0.7 | 2.1 | 0.7 | -0.9 | 0.360 |
| *LED (mg/day)* | 173.5 | 129.7 | 189.5 | 187.9 | -0.3 | 0.740 |
| *GDS-15* | 2.7 | 2.4 | 3.3 | 2.9 | -1.2 | 0.221 |
| *MoCA*^†^ | 25.2 | 3.7 | 25.1 | 3.5 | -0.4 | 0.707 |
|  | n | % | n | % | χ^2^ | p |
| *Sex (male)* | 74 | 67 | 26 | 59 | 0.9 | 0.355 |

NART = National Adult Reading Test, MDS-UPDRS III = Movement Disorders Society-Unified Parkinson’s Disease Rating Scale Part III, LED = Levodopa equivalent dose, GDS-15 = Geriatric Depression Scale, MoCA = Montreal Cognitive Assessment.

† For MoCA, Completers n= 98, Non-completers n= 41.
